# Clinical features and risk factors of neurological involvement in Sjögren’s syndrome

**DOI:** 10.1186/s12868-018-0427-y

**Published:** 2018-04-27

**Authors:** Wenjing Ye, Siyan Chen, Xinshi Huang, Wei Qin, Ting Zhang, Xiaofang Zhu, Xiaochun Zhu, Chongxiang Lin, Xiaobing Wang

**Affiliations:** 1 Rheumatology Department, The First Affiliated Hospital of Wenzhou Medical University, Wenzhou, China; 2 Rheumatology Department, Ruian People’s Hospital, Wenzhou, China; 3 Neurology Department, The First Affiliated Hospital of Wenzhou Medical University, Wenzhou, China; 4 Stomatological Department, The First Affiliated Hospital of Wenzhou Medical University, Wenzhou, China

**Keywords:** Sjögren’s syndrome, Cross-sectional study, Neurological complication, Risk factor, Logistic model

## Abstract

**Background:**

To investigated distinct manifestations of Sjögren’s syndrome (SS) patients with neurological complications and the potential risk factors associated with neurological complications in SS, and to produce a disease evaluation and neurological involvement prediction for SS.

**Methods:**

566 patients who fulfilled the 2002 classification criteria for SS from the Rheumatology Department of the First Affiliated Hospital of Wenzhou Medical University were included in the cross-sectional study. Clinical, immunological and histological characteristics were surveyed, and potential risk factors for neurological complications were examined by multivariate analysis.

**Results:**

Among 566 SS patients, 184 (32.5%) patients had neurological involvement, with more than 10% got limbs pain, limbs numbness and cerebral infarction, respectively. Of these 184 SS patients with neurological complications, secondary SS (sSS) patients had a higher prevalence of peripheral nervous system (PNS) involvement than primary SS (pSS) patients (31.1 vs. 19%). And sSS patients showed higher total ESSPRI score and higher prevalence of xerostomia and low C3, C4 levels with more liver, articular involvement and saliva gland atrophy, and more severe lymphocyte infiltration in salivary glands than pSS patients. As for the specific factors associated with neurological involvement, low C3 level were found to be significant in pSS or sSS patients who were younger 50 year old, and ANA positivity, cardiac involvement, saliva gland atrophy were demonstrated to be associated in elder pSS patients. And xerophthalmia was found to be associated in sSS patients.

**Conclusion:**

Low complement (C3) levels, xerophthalmia, ANA positive, cardiac involvement and labial salivary gland histological result were good ways to predict neurological complications in different subgroups of SS, which might provide insight into better clinical decision-making, especially at early stages of the disease.

## Background

Sjögren’s syndrome (SS) is a chronic autoimmune disease characterized by autoantibody secretion and lymphocytic infiltrates of exocrine glands leading to xerophthalmia and xerostomia [1]. Sjögren’s syndrome is considered secondary (sSS) when it presents with associated autoimmune disorders, otherwise it’s considered primary Sjögren’s syndrome (pSS) [2]. It impacts 0.1–0.6% of the general adult population with a female to male ratio of 9:1 [3]. Besides dry eyes and dry mouth, pSS covers a broad spectrum of extraglandular clinical presentations, including vasculitis, arthralgia, renal tubular acidosis, pulmonary involvement, immunological abnormalities and neuropathy [4, 5].

Neurological disorders of SS including peripheral neuropathy and central neuropathy were investigated since the 1980s [6], however, their prevalence are inconstant in different studies. It’s documented that 10–20% of SS patients had peripheral nervous system (PNS) involvement [7]. However, the prevalence of central nervous system (CNS) manifestations of SS ranged from 2.5 to 60% [8], which may due to the lack of unified definition of CNS involvement in SS [9, 10]. Therefore, CNS and PNS complications are common but varied in SS. Furthermore, the association between clinical/serological manifestations and nervous system involvement remains unclear. To date no seroimmunological profiles had been shown as pathognomonic neither for neurological involvement in SS. The aim of this study was to evaluate the prevalence and symptoms of neurological complications in a population of patients with SS and to investigate the potential risk factors for neurological involvement to facilitate a comprehensive disease evaluation and prognosis prediction for Chinese SS patients.

## Methods

### Study population and clinical data

A total of 566 patients who fulfilled the 2002 classification criteria [8] for SS from the Rheumatology department of the First Affiliated Hospital of Wenzhou Medical University between January 1, 2013 and February 28, 2017 were enrolled in this study. The study was approved by the Ethical Committee of the First Affiliated Hospital of Wenzhou Medical University (approval # 16024). The study design conformed to current National Health and Family Planning Commission of China ethical standards, with written informed consent provided by all patients. sSS patients were diagnosed with systemic lupus erythematosus [11], systemic sclerosis [12], rheumatoid arthritis [13], inflammatory myopathies [14], mixed connective tissue disease [15], or primary biliary cirrhosis [16] according to existing classification criteria used in clinical practice. Clinical data as age of disease onset, age at diagnosis, the duration of the disease, oral and ocular dryness, constitutional symptoms, joints, skin, pulmonary, kidney, vasculitis, gastrointestinal tracts and endocrine involvement were collected.

### Assessment of nervous system involvement

Peripheral neuropathy includes painful sensory neuropathy, sensory ataxic neuropathy, pure sensory trigeminal neuropathy, axonal sensorimotor polyneuropathy, mononeuritis multiplex, multiple cranial neuropathies, radiculoneuropathy and autonomic neuropathy. Involvement of peripheral nervous system was diagnosed by medical history, physical examination and electromyography. Involvement of central nervous system was confirmed by case history brain computed tomography, cranial MRI, electromyography and psychiatric records. And the presentations we collect including headache, dizziness, physical pain, epilepsy, cognitive disorder, disturbance of consciousness, emotional problem, cerebral hemorrhage, cerebral infarction, demyelination, vascular stenosis and occlusion, aging brain, cerebral tumor, leukoaraiosis and nerve injury. Depression was diagnosed by psychiatrists.

### Assessment of exocrine gland involvement and disease activity

All patients underwent a thorough review of medical history, physical examination and a series of assessments to evaluate the systemic condition. Xerostomia was diagnosed by testing timed whole unstimulated salivary flow and the histopathological result of labial salivary gland biopsy (LSGB). A lymphocytic focus score of ≥ 1 as a positive biopsy with more than 50 lymphocytes per 4 mm^2^, based on the classification described previously [17]. Xerophthalmia was confirmed by an ophthalmologist by examining Schirmer’s test [16], breakup time of tear film [18] and cornea fluorescent pigmentation [19].

Besides routine blood test, immunological tests including ANA, anti-Ro antibodies, anti-SSA antibodies, anti-SSB antibodies, rheumatoid factor, C3, C4, IgG, IgA, and IgM levels were performed using commercial techniques. Additionally, all patients were tested for acute inflammatory factors comprising erythrocyte sedimentation rate (ESR), and C-reactive protein (CRP) levels.

Disease manifestations were scored with the ESSDAI summating the scores achieved per organ domain. The ESSDAI scores 12 organ domains on the severity of involvement, ranging from 0 to 3 points. And each patient was asked to assess the severity of dryness, fatigue, and pain over the preceding 2 weeks, each on a 10-point Likert scale. The average of the three items was regarded as ESSPRI value [20].

### Statistical analysis

Continuous data were expressed as mean ± standard deviation, and counts and percentages were indicated for the categorical variables. Nonparametric Wilcoxon signed-rank test was used for paired comparison and Fisher’s exact tests used for categorical data as appropriate. To examine correlations between risk factors and neuropathy, univariate analyses were used, firstly based on biological plausibility and literature review. Variables with a *P* < 0.05 in univariate analysis were then included in a multivariate analysis using logistic regression. Statistical significance was set at *P* < 0.05. All analyses were conducted using R v3.3.2 statistical software packages.

## Results

### The characteristics of Sjögren’s syndrome patients with or without neurological involvement

The patient cohort comprised 566 individuals, with 415 pSS and 151 sSS patients. Demographic, clinical, histological, immunological, inflammatory features and outcome measures data collected from 415 pSS were presented in Table 1. The female to male ratio is 10:1. 290 (69.88%) pSS patients had no neurological involvements and 125 (30.12%) with neurological involvement. Most patients presented to the hospital in their 50s for the first interview, with an average disease course of approximate 4 years. Compared with pSS patients without neurological involvement, those with neurological involvement showed much higher total ESSDAI score with more cardiac and constitutional domain involvements, but less glandular domain involvement. It is consistent with a lower prevalence of xerostomia in this subgroup. However, no significant difference in total ESSPRI scores was found between the two groups. pSS patients with neurological involvement showed a higher prevalence of ANA antibody and hypergammaglobulinemia, but reduced level of C3 (*P *< 0.05). No significant differences in Anti-SSA/SSB positive was found between the two subgroups (*P *> 0.05). Although pSS patients with neurological involvement seemed to use glucocorticoid more often with higher dose and longer duration, the differences didn’t reach to the statistical significance (*P *< 0.05, Table 1). There are 92 (60.93%) sSS patients had no neurological involvements and 59 (39.07%) with neurological involvement. The comparison between with and without neurological involvement were not analyzed as sSS cases had different primary diseases with distinctive etiology and mechanism.

**Table 1:** Demographic, clinical, histological, immunological, inflammatory features and outcome measures of Chinese primary Sjögren’s syndrome patients with or without neurological involvement

	Without NP involvement	With NP involvement	*P* value
	290	125	
Age (years)	51.50 ± 13.73	52.81 ± 14.95	0.387
Sex, female, n (%)	28 (9.66%)	16 (12.80%)	0.34
Disease duration (months)	3.99 ± 6.01	4.30 ± 5.57	0.642
Hypertension, n (%)	57 (19.66%)	29 (23.20%)	0.414
Diabetes mellitus, n (%)	22 (7.59%)	12 (9.60%)	0.493
Hyperlipemia, n (%)	114 (39.31%)	37 (29.60%)	0.059
Xerostomia, n (%)	237 (81.72%)	88 (70.40%)	0.010
Xerophthalmia, n (%)	80 (27.59%)	44 (35.20%)	0.120
ESSDAI	6.60 ± 2.22	8.81 ± 3.45	< 0.001
ESSPRI	2.87 ± 1.51	2.97 ± 1.46	0.459
Constitutional domain involvement	65 (22.41%)	42 (33.60%)	0.017
Glandular domain involvement	189 (65.17%)	47 (37.60%)	< 0.001
Cardiac involvement, n (%)	133 (45.86%)	77 (61.60%)	0.003
LSGB, Lymphocytic focus ≥ 1	192 (66.21%)	76 (60.80%)	0.291
ANA positivity, n (%)	254 (87.59%)	120 (96.00%)	0.008
Anti-SSA/Ro60 positive, n (%)	214 (75.09%)	97 (78.23%)	0.494
Anti-Ro52 positive, n (%)	177 (62.11%)	79 (63.20%)	0.833
Anti-SSB positive, n (%)	134 (47.02%)	50 (40.00%)	0.188
Hypergammaglobulinemia (> 16 g/L), n	186 (64.14%)	120 (100.00%)	< 0.001
Low C3 level (< 0.9 g/L)	90 (31.03%)	54 (46.15%)	0.004
Low C4 level (< 0.1 g/L)	11 (4.17%)	6 (5.31%)	0.624
RF positive, n (%)	178 (61.38%)	59 (52.21%)	0.093
ESR (mm/h)	35.71 ± 23.84	32.43 ± 25.31	0.232
CRP (mg/L)	10.23 ± 21.68	12.76 ± 24.51	0.325
Glucocorticoid use	171 (58.97%)	81 (66.39%)	0.158
Moderate to high dose	46 (15.86%)	24 (19.20%)	0.405
Continuous use for > 1 year	81 (27.93%)	38 (30.40%)	0.610

Disease duration measured from the day diagnosed; moderate- to high-dose glucocorticoid: > 1 mg/kg/d prednisone; glucocorticoid use at any time for > 2 week

*NP* neurological, *ESSDAI* European League Against Rheumatism Sjögren’s syndrome disease activity index, *ESSPRI* European League Against Rheumatism Sjögren’s syndrome patient reported index, *ANA* antinuclear antibodies, *C3* complement component 3, *C4* complement component 4, *CRP* C-reactive protein, *ESR* erythrocyte sedimentation rate, *LSGB* labial salivary gland biopsy, *RF* rheumatoid factor

### The characteristics of neurological involvement in Sjögren’s syndrome patients

As shown in Table 2, the various manifestations of neurological involvements were categorized into five subgroups: PNS presentations, neuropsychiatric symptoms, unspecific neurological complaints, paroxysmal disease, and CNS radiological results. The prevalence of limbs pain, limbs numbness and cerebral infarction were all higher than 10%. Meanwhile, the occurrences of trigeminal neuralgia, epilepsy, cerebral hemorrhage, angiostegnosis, cerebral tumor and leukoaraiosis were comparatively low. Of note, there were 39 patients with neurological involvement had sleep problem as well.

**Table 2:** Features of neurological involvement in Sjögren’s syndrome patients

Neurological involvement	Numbers	Percentage (%)
*PNS presentations*		
Limbs pain	35	0.190
Limbs numbness	25	0.136
Anaesthesia	8	0.043
Trigeminal neuralgia	1	0.005
*Neuropsychiatric symptoms*		
Depression	10	0.054
Cognitive dysfunction	4	0.022
*Unspecific neurological complaints*		
Headache	15	0.082
Dizziness	16	0.087
Muscular weakness	11	0.060
*Paroxysmal disease*		
Epilepsy	3	0.016
*CNS radiological results*		
Cerebral hemorrhage	2	0.011
Cerebral infarction	35	0.190
Demyelination	5	0.027
Angiostegnosis	3	0.016
Aging brain	11	0.060
Cerebral tumor	3	0.016
Leukoaraiosis	3	0.016

*PNS* peripheral nervous system, *CNS* central nervous system

### Subtypes of neurological involvement in Sjögren’s syndrome patients

Among 415 pSS patients, there were 125 of them had neurological involvement with 79 PNS involvement and 63 CNS involvement. 59 out of 151 sSS patients got neurological involvement. And sSS had a higher prevalence of PNS involvement than pSS (31.13 vs. 19.04%, *P *< 0.05), although no significant difference in CNS involvement (13.91 vs. 15.18%). Most of the sSS cases with neurological involvement were secondary to systemic lupus erythematosus (SLE) or rheumatoid arthritis (RA) (Table 3).

**Table 3:** Subtypes of neurological involvement in Sjögren’s syndrome patients

	PNS involvement	CNS involvement
pSS (n = 125)	79	63
sSS (n = 59)	47	21
Rheumatoid arthritis	13	5
Systemic lupus erythematosus	28	13
Systemic sclerosis	1	0
Dermatomyositis/myositis	3	0
Primary biliary cirrhosis	0	3
Mixed connective tissue disease	1	1

### Features of Sjögren’s syndrome patients with neurological involvement

As shown in Table 4, sSS showed higher total ESSPRI score and higher prevalence of xerostomia and low C3, C4 levels with more liver, articular involvement and saliva gland atrophy, while pSS had a higher level of albumin. And sSS showed more severe lymphocyte infiltration in salivary glands than pSS. There were 6 antiphospholipid syndrome cases were found in sSS but none in pSS.

**Table 4:** Analysis of multiple features of 184 Chinese Sjögren’s syndrome patients with neurological involvement

	pSS	sSS	*P* value
	125	59	
Age (years)	52.81 ± 14.95	47.93 ± 16.22	0.084
Sex, female, n (%)	109 (87.20%)	56 (94.90%)	0.126
Disease duration (months)	51.57 ± 66.84	46.07 ± 68.73	0.955
Xerostomia, n (%)	88 (70.40%)	50 (84.70%)	0.036
Xerophthalmia, n (%)	44 (35.20%)	22 (37.30%)	0.783
ESSDAI	7.84 ± 2.83	8.81 ± 3.45	0.129
ESSPRI	2.77 ± 1.34	3.38 ± 1.63	0.038
Liver dysfunction, n (%)	41 (33.60%)	28 (49.10%)	0.047
Articular involvement, n (%)	46 (36.80%)	40 (67.80%)	< 0.001
LSGB, lymphocytic focus ≥ 1	76 (60.80%)	50 (84.70%)	0.001
Saliva gland atrophy	78 (70.30%)	46 (86.80%)	0.021
Albumin levels (g/L)	37.35 ± 5.73	34.12 ± 5.94	< 0.001
Low C3 level (< 0.9 g/L)	54 (46.20%)	36 (65.50%)	0.018
Low C4 level (< 0.1 g/L)	6 (5.30%)	11 (20.80%)	0.005
Antiphospholipid syndrome	0	6(10.17%)	< 0.001

*NP* neurological, *ESSDAI* European League Against Rheumatism Sjögren’s syndrome disease activity index, *ESSPRI* European League Against Rheumatism Sjögren’s syndrome patient reported index, *ANA* antinuclear antibodies, *C3* complement component 3, *C4* complement component 4, *CRP* C-reactive protein, *ESR* erythrocyte sedimentation rate, *LSGB* labial salivary gland biopsy, *RF* rheumatoid factor

### Specific factors associated with neurological involvement in Sjögren’s syndrome

A series of features commonly used in clinical practice were selected first by univariate analysis and then logistic regression analysis as potential risk factors for neurological involvement in SS. By univariate analysis a series of variables were found to be associated with neurological involvement, as shown in Table 5. And their independent risks for neurological involvement were further tested by multivariate analysis. As age is a key factor on neurological involvement, we did a stratification analysis by separate the cohort into two subgroups. There were 248 patients younger than 50, and 318 were the opposite (including 50 years old). We found that male, low C3 level presented significant associations with neurological involvement in pSS patients who were younger than 50 year old, while ANA positivity, cardiac involvement, saliva gland atrophy were found significant in those older than 50 year old (Table 6). Xerophthalmia and low C3 level were demonstrated to be associated with neurological involvement in the younger subgroup of sSS patients, and xerophthalmia level also presented in the older subgroup (Table 6).

**Table 5:** Univariate and multivariate analysis of factors associated with neurological involvement in primary and secondary Sjögren’s syndrome

Independent variables	Univariate analysis OR (95% CI) *P* value	
	pSS	sSS
*Age < 50*		
Sex (male)	4.55 (1.27, 16.29) 0.020	–
ESSDAI	1.47 (1.26, 1.72) < 0.001	1.27 (1.05, 1.53) 0.0136
Xerophthalmia, n (%)		3.25 (1.03, 10.23) 0.0440
Low C3 level (< 0.9 g/L)	2.10 (1.06, 4.13) 0.032	3.83 (1.31, 11.24) 0.0143
Low C4 level (< 0.1 g/L)	–	3.50 (1.02, 12.00) 0.0463
RF positive, n (%)	–	0.32 (0.12, 0.87) 0.0251
ESR (mm/h)	0.98 (0.96, 1.00) 0.038	–
*Age ≥ 50*		
Constitutional domain involvement	1.96 (1.08, 3.55) 0.027	–
ESSDAI	1.52 (1.34, 1.72) < 0.001	1.44 (1.16, 1.80) 0.001
ANA positivity, n (%)	4.50 (1.02, 19.83) 0.047	–
Cardiac involvement, n (%)	2.28 (1.21, 4.31) 0.010	–
Xerophthalmia, n (%)	1.82 (1.02, 3.27) 0.044	4.82 (1.56, 14.95) 0.006
Low C3 level (< 0.9 g/L)	1.83 (1.01, 3.30) 0.046	–
Saliva gland atrophy	0.38 (0.19, 0.74) 0.004	–
ESR (mm/h)	–	0.98 (0.96, 1.00) 0.0336

*NP* neurological, *ESSDAI* European League Against Rheumatism Sjögren’s syndrome disease activity index, *ESSPRI* European League Against Rheumatism Sjögren’s syndrome patient reported index, *ANA* antinuclear antibodies, *C3* complement component 3, *C4* complement component 4, *CRP* C-reactive protein, *ESR* erythrocyte sedimentation rate, *LSGB* labial salivary gland biopsy, *RF* rheumatoid factor

**Table 6:** Multivariate analysis of factors associated with neurological involvement in primary and secondary Sjögren’s syndrome

Independent variables	Multivariate analysis OR (95% CI) *P* value	
	pSS	sSS
*Age < 50*		
Sex (male)	4.56 (1.15, 20.01) 0.013	
Low C3 level (< 0.9 g/L)	2.21 (1.04, 4.82) 0.026	3.33 (1.92, 8.03) 0.021
Xerophthalmia, n (%)		3.03 (1.08, 7.45) 0.030
*Age >= 50*		
ANA positivity	5.52(1.42, 37.28) 0.037	
Cardiac involvement	2.38 (1.18, 5.06) 0.013	
Saliva gland atrophy	0.28 (0.13, 0.59) 0.001	
Xerophthalmia, n (%)		4.07 (1.25, 14.35)0.012

*NP* neurological, *ESSDAI* European League Against Rheumatism Sjögren’s syndrome disease activity index, *ESSPRI* European League Against Rheumatism Sjögren’s syndrome patient reported index, *ANA* antinuclear antibodies, *C3* complement component 3, *C4* complement component 4, *CRP* C-reactive protein, *ESR* erythrocyte sedimentation rate, *LSGB* labial salivary gland biopsy, *RF* rheumatoid factor

## Discussion

This study is to investigate the neurological involvement in a Chinese SS cohort. The prevalence of peripheral and central nervous system are about 19 and 15.2% respectively in pSS, which lied in the wide ranges came from various studies [21, 22]. However, sSS seemed to complicate with more PNS manifestations (31.1%). Most of our sSS were secondary to SLE and RA. It’s been reported the prevalence of neuropsychiatric syndromes in SLE at time of diagnosis with SLE were 28% [23], but the estimate incidence of neurological symptoms in RA were high up to 70% when mood disorders are included [24]. In our cohort, neuropsychiatric symptoms like depression is not uncommon, which should arouse more attention. Among various manifestations of neurological involvement, limbs pain, limbs numbness and cerebral infarction were most frequently seen in more than a third of the cohort which suggesting an ischemic mechanism and a vasculitis process in SS. Epilepsy had been described in pSS, but is not well characterized [7]. We also found 3 cases with epilepsy as their initial symptoms and were then diagnosed by neuropathists. When compare the differences between pSS and sSS, we found the later have higher frequencies of xerostomia, articular and saliva gland involvement and low complement prevalence. Given the wide involvement of neurological involvement in SS, tight control of neurological risk factors is needed for better prognosis.

For potential risk factors for neurological involvement, low C3 level was found to be associated in younger pSS patients, and ANA positivity, cardiac involvement, saliva gland atrophy were demonstrated to be associated in elder pSS patients. It revealed that cardiac event accumulates with age and plays a key role in neurological involvement. For example, a marked increase in the risk of stroke was found after acute myocardial infarction stroke revealed its strong association with high cardiovascular risk [25]. Saliva gland atrophy was confirmed by histology of LSGB. Significant lymphocytic in filtration in the LSGB, defined as a focus score (FS) ≥ 1, has a preponderant role in both AECG and ACR classifications and is a promising tool for prognosis [26]. It and has sensibility comparable to scintigraphy and sialography and could not be substituted by ultrasound for all patients [27]. However, the sensitivity and specificity of commonly seen saliva gland atrophy for SS remain unclear. Here, we reveal the potential relationship between saliva gland atrophy and neurological involvement in pSS, especially meaningful in the elder, while the pathogenesis to be further investigated. ANA positivity has been found to be associated with uveitis risk in Juvenile idiopathic arthritis [28] and is considered as a significant predictors of cardiovascular events and mortality in both those with and those without rheumatic diseases, which hints the its potential role in neurological involvement [29].

Furthermore, xerophthalmia were found to be associated with neurological involvement in both younger and older sSS patients, but low C3 level was only connected with those younger sSS patients. As is known, the complement system is a major component of the innate immune system, is becoming increasingly recognized as a critical participant in the acute inflammatory pathophysiology of acquired brain or spinal cord injury [30, 31]. C3 represents the central molecule of the complement cascade [32]. Low level of C3 in SS patients’ serum which suggesting the excessive activation and consumption of complement system, was revealed to be an independent risk factor for neurological involvement both in pSS and sSS patients who were less than 50 years old. Our finding further supports the role of complement system in immunological associated neurological lesion. Also, the different risk factors of neurological involvement in the two age subgroups suggested distinctive mechanisms of pathogenesis behind them.

The broad spectrums of clinical profiles, different classification criteria and ethnic diversity have posed challenges to a comprehensive and accurate appraisal of SS. In our study it’s demonstrated that low C3 level is a good to predictor for neurological complications in younger SS, both in primary and secondary SS, which might provide insight into better clinical decision-making, especially at early stages of the disease.

However, limitation of this study should be considered, that we included a homogenous group of participants from one center, which might provide compelling results. As it is a cross-sectional study, is limited to correlation analysis and unable to support strong causal conclusions. And traditional risk factors like arteriosclerosis, age, hypertension, therapeutic side effects, were not adjusted in the present study, which might also play a key role in the development of the neurological complications in SS. Therefore, to further evaluate the role of complement neurological complications in SS, more data from heterogeneous SS patients with consecutive follow-up are highly recommended.

## Conclusions

Low complement (C3) levels, xerophthalmia, ANA positive, cardiac involvement and labial salivary gland histological result were good ways to predict neurological complications in different subgroups of SS, which might provide insight into better clinical decision-making, especially at early stages of the disease.

## Abbreviations

SS: Sjögren’s syndrome
sSS: secondary Sjögren’s syndrome
pSS: primary Sjögren’s syndrome
PNS: peripheral nervous system
C3/C4: complement 3/complement 4
CNS: central nervous system
LA: leukoaraiosis
LSGB: labial salivary gland biopsy
ESR: erythrocyte sedimentation rate
CRP: C-reactive protein
RF: rheumatoid factor
ANA: antinuclear antibodies
EULAR: European League Against Rheumatism
ESSDAI: European League Against Rheumatism Sjögren’s syndrome disease activity index
ESSPRI: European League Against Rheumatism Sjögren’s syndrome patient reported index
